# UbiSite: incorporating two-layered machine learning method with substrate motifs to predict ubiquitin-conjugation site on lysines

**DOI:** 10.1186/s12918-015-0246-z

**Published:** 2016-01-11

**Authors:** Chien-Hsun Huang, Min-Gang Su, Hui-Ju Kao, Jhih-Hua Jhong, Shun-Long Weng, Tzong-Yi Lee

**Affiliations:** Department of Computer Science and Engineering, Yuan Ze University, Taoyuan, 320 Taiwan; Ministry of Health & Welfare, Tao-Yuan Hospital, Taoyuan, 320 Taiwan; Department of Obstetrics and Gynecology, Hsinchu Mackay Memorial Hospital, Hsin-Chu, 300 Taiwan; Mackay Junior College of Medicine, Nursing and Management , Taipei, 112 Taiwan; Department of Medicine, Mackay Medical College, New Taipei City, 252 Taiwan; Innovation Center for Big Data and Digital Convergence, Yuan Ze University, Taoyuan, 320 Taiwan

**Keywords:** Ubiquitin conjugation, Ubiquitylation, Substrate motif, Position-specific scoring matrix

## Abstract

**Background:**

The conjugation of ubiquitin to a substrate protein (protein ubiquitylation), which involves a sequential process – E1 activation, E2 conjugation and E3 ligation, is crucial to the regulation of protein function and activity in eukaryotes. This ubiquitin-conjugation process typically binds the last amino acid of ubiquitin (glycine 76) to a lysine residue of a target protein. The high-throughput of mass spectrometry-based proteomics has stimulated a large-scale identification of ubiquitin-conjugated peptides. Hence, a new web resource, UbiSite, was developed to identify ubiquitin-conjugation site on lysines based on large-scale proteome dataset.

**Results:**

Given a total of 37,647 ubiquitin-conjugated proteins, including 128026 ubiquitylated peptides, obtained from various resources, this study carries out a large-scale investigation on ubiquitin-conjugation sites based on sequenced and structural characteristics. A TwoSampleLogo reveals that a significant depletion of histidine (H), arginine (R) and cysteine (C) residues around ubiquitylation sites may impact the conjugation of ubiquitins in closed three-dimensional environments. Based on the large-scale ubiquitylation dataset, a motif discovery tool, MDDLogo, has been adopted to characterize the potential substrate motifs for ubiquitin conjugation. Not only are single features such as amino acid composition (AAC), positional weighted matrix (PWM), position-specific scoring matrix (PSSM) and solvent-accessible surface area (SASA) considered, but also the effectiveness of incorporating MDDLogo-identified substrate motifs into a two-layered prediction model is taken into account. Evaluation by five-fold cross-validation showed that PSSM is the best feature in discriminating between ubiquitylation and non-ubiquitylation sites, based on support vector machine (SVM). Additionally, the two-layered SVM model integrating MDDLogo-identified substrate motifs could obtain a promising accuracy and the Matthews Correlation Coefficient (MCC) at 81.06 % and 0.586, respectively. Furthermore, the independent testing showed that the two-layered SVM model could outperform other prediction tools, reaching at 85.10 % sensitivity, 69.69 % specificity, 73.69 % accuracy and the 0.483 of MCC value.

**Conclusion:**

The independent testing result indicated the effectiveness of incorporating MDDLogo-identified motifs into the prediction of ubiquitylation sites. In order to provide meaningful assistance to researchers interested in large-scale ubiquitinome data, the two-layered SVM model has been implemented onto a web-based system (UbiSite), which is freely available at http://csb.cse.yzu.edu.tw/UbiSite/. Two cases given in the UbiSite provide a demonstration of effective identification of ubiquitylation sites with reference to substrate motifs.

**Electronic supplementary material:**

The online version of this article (doi:10.1186/s12918-015-0246-z) contains supplementary material, which is available to authorized users.

## Background

In 1975, Goldstein et al. [1] discovered ubiquitin, which is a small protein of approximately 8.5 kDa and is composed of 76 amino acids [2]. The attachment of mono-ubiquitin or poly-ubiquitin chains to proteins at particular lysines is a critical post-translational modification (PTM), which is called protein ubiquitylation or ubiquitination [3, 4]. Protein ubiquitylation, involving a sequential process – E1 activation, E2 conjugation and E3 ligation enzyme, is important for modulating various cellular functions, such as transcriptional regulation, signal transduction, development, apoptosis, endocytosis, and cell proliferation [5–7]. These three major classes of enzymes have critical roles in promoting and controlling the activities that are involved in the process of protein ubiquitylation system. In particular, the E3 ligases recognize a specific substrate protein and then catalyze the attachment of ubiquitin to the target lysine of a protein [8, 9]. The HECT domain of E3 ligases plays prominent roles in trafficking, immune response, and several signaling pathways that regulate cellular growth and proliferation [10]. As illustrated in Figure S1 (Additional file 1), the HECT domain consists of two major components: a N-terminal N-lobe that interacts with the E2, and a C-terminal C-lobe which contains the active-site cysteine forming the thioester linkage with ubiquitin [11–13]. The conserved HECT domain is located at the C-terminus of these enzymes, whereas their N-terminal domains are diverse and mediate substrate targeting.

With the biological importance of protein ubiquitylation, the high-throughput of mass spectrometry-based proteomics has stimulated a large-scale identification of ubiquitin-conjugated peptides [14–16]. Thus, numerous databases [9, 17–20] of protein ubiquitylation sites have been developed to date, owing to the regulatory significance of ubiquitin in cellular processes. In addition, the labor-intensive experiment in site-specific identification of ubiquitin-conjugated peptides motivated an increasing number of computational methods [14, 16, 21–25] developed for the identification of ubiquitylation sites. As the number of experimentally verified ubiquitylation sites is increasing, the development of new bioinformatics tools that can be applied to large-scale proteome data is required. Recently, an ensemble classifier was developed to identify ubiquitylation sites based on lysines [26]. The WPNNA (weighted passive nearest neighbor algorithm) classifier for identifying ubiquitylation sites based on hybrid features has also been proposed [27]. The UbiProber [28], which integrates key position and amino acid residue features, was designed to predict both general and species-specific ubiquitylation sites. Additionally, independent testing has demonstrated that the combined model improves the area under the operating characteristic curve of the receiver (AUC) by ~15 %. Although the fact that the accuracy and stability of these prediction tools have been demonstrated, room typically exists to improve its predictive power. Moreover, as more experimentally confirmed ubiquitylation sites become available, the lack of methods for characterizing the substrate site specificities based on large-scale datasets is serious.

An increasing number of site-specific ubiquitylated peptides obtained from mass spectrometry-based proteomics has stimulated this work to characterize and identify substrate sites of ubiquitin conjugation based on large-scale dataset. Experimentally confirmed ubiquitin-conjugated peptides were mainly collected from several online resources, including hCKSAAP_UbiSite [22], dbPTM [19, 20] and mUbiSiDa [18]. Not only are sequence-based features, such as amino acid composition (AAC), amino acid pairwise composition (AAPC), positional weighted matrix (PWM) and position-specific scoring matrix (PSSM) assessed, but also the effectiveness of solvent-accessible surface area (SASA) or secondary structure is examined. A best feature for the prediction of ubiquitylation sites was determined based on five-fold cross-validation. Additionally, a motif discovery tool, MDDLogo [29], was adopted to characterize the potential substrate motifs of ubiquitylation sites and to generate a two-layered prediction model by integrating the MDDLogo-identified substrate motifs. The independent testing showed that the two-layered SVM model could provide a promising accuracy (73.69 %) and perform better than other prediction tools. Finally, the two-layered prediction model was adopted to implement a web-based system (UbiSite) for providing effective assistance to researchers who are interested in large-scale proteome data.

## Methods

### Collection and preprocessing of training dataset

Experimentally validated ubiquitin-conjugated peptides were mainly obtained from several online resources, including hCKSAAP_UbiSite [22], dbPTM [19, 20], and mUbiSiDa [18]. As presented in Table 1, totally 6118 ubiquitylated lysines were taken from 2500 ubiquitylated proteins. The dataset associated with protein ubiquitylation was also obtained from version 3.0 of dbPTM, which is a comprehensive database of protein post-translational modifications that has accumulated 23,949 ubiquitylated lysines from 6259 proteins. Additionally, a large-scale ubiquitin-conjugated peptides (110,695 ubiquitylated lysines in mammals) were obtained from mUbiSiDa, which is a comprehensive database containing 35,494 ubiquitylated proteins. After redundant data were removed, 37,647 ubiquitylated proteins, consisting of 128,026 ubiquitylated lysines, were regarded as the training dataset.

**Table 1:** Data statistics in the construction of training dataset and independent testing dataset

Data set	Data resource	Number of ubiquitylated proteins	Number of ubiquitylated lysines	Number of non-ubiquitylated lysines
Training set	hCKSAAP_UbiSite	2500	6118	6118
	dbPTM 3.0	6259	23,949	228,441
	mUbiSiDa	35,494	110,695	1,217,977
	Combined non-redundant data	37,647	128,026	1,317,734
	Non-homologous data (sequence identity ≦ 30 %)	4828	5438	12,663
Independent testing set	CPLM 2.0	32,429	139,950	1,109,432
	Non-homologous data (sequence identity ≦ 30 %)	2894	3732	10,664

To construct positive data of training dataset, herein, a window of length 2*n* + 1 was used to extract sequence fragments that were centered at the ubiquitylated lysine (K) residue and contained *n* upstream and *n* downstream flanking amino acids. Given 37,647 experimentally verified ubiquitylated proteins, the sequence fragments that contained window length of 2*n* + 1 amino acids and were centered at the lysine residue without an annotation of ubiquitylation were regarded as the negative data of training dataset (non-ubiquitylated lysines). According to another work [14] and the preliminary evaluation using windows of various lengths, a window size of 13 (*n* = 6) maximizes the accuracy in the prediction of ubiquitylation sites. Based on a window size of 13, the training dataset contains 128,026 positive data and 1,317,734 negative data.

As for binary classification, the performance of the predictive models may be overestimated or underestimated owing to the homologous sequences in positive and negative datasets. In order to obtain a reasonable prediction performance, the homologous sequences should be removed from the training dataset by using CD-HIT [30] program. Additionally, an analysis of sequence fragments indicates that, owing to the incompleteness of available information about experimentally validated ubiquitylation sites, some negative data may be homologous to positive data in the training dataset, potentially resulting in false positive or false negative predictions. Accordingly, CD-HIT was used again by running cd-hit-2d across positive and negative training data with 50 % sequence similarity to filter out the homologous sequences in negative data. Table S1 (Additional file 2) presents statistics concerning the data after the homologous sequences had been removed using CD-HIT, based on various values of sequence identity. After homologous sequences with 30 % sequence identity had been filtered out using cd-hit and psi-cd-hit, the training dataset comprised 5438 positive sequences and 12,663 negative sequences.

### Features extraction and encoding

In this work, several sequence-based features, including amino acid composition (AAC), amino acid pairwise composition (AAPC), positional weighted matrix (PWM), and position-specific scoring matrix (PSSM), were examined. Additionally, the solvent-accessible surface area (SASA) and secondary structure around the ubiquitylation sites were also investigated. Amino acid sequences with a lysine in the center were individually extracted from positive and negative training sets using a window of length 2*n* + 1, where *n* was set to six. To transform the amino acid sequence into a numeric vector for model construction, a basic encoding method, namely 20-dimensional binary coding (20D), was adopted. For instance, Alanine (A) was encoded as “10000000000000000000”, and Cysteine (C) was encoded as “01000000000000000000”. The number of dimensions for 20D feature was (2*n* + 1) × 20 to represent the flanking amino acids surrounding the ubiquitylation sites. The training data contained *k* vectors {*x*_*i*_, *i* = 1, 2 …, *k*} corresponding to the *k* fragment sequences. The labels +1 and −1 were used to mark the positive and negative data classes, respectively, for each numeric vector.

For the coding of amino acid composition, each sequence fragment was encoded as a 21-dimensional vector {*x*_*i*_, *i* = 1,…,*21*} that comprised 20 types of amino acid and an non-existing residue, which specify the number of occurrences of the 20 types of amino acid normalized with the total number of residues in a sequence fragment. For the coding of amino acid pair composition, a 21x21-dimensional matrix {*x*_*ij*_, *i ,j* = 1, …, *21*} was used to encode each sequence fragment. Each element *x*_*ij*_ specifies the number of occurrences of amino acid pairs normalized with the total number of amino acid pairs in a sequence fragment. By referring to the SulfoSite method [31], PWM was determined by calculating the occurrence rate of 20 amino acids surrounding a substrate sites, and was utilized in encoding the fragment sequences. Each sequence fragment of the training dataset was represented by a matrix of *(*2*n* + 1*) × w* elements, where *w* stands for 21 elements including 20 types of amino acids and one for the non-existing residue.

In the viewpoint of protein sequence evolution, several amino acid residues of a protein can be mutated without changing its core structure or functional domain, and two proteins may have similar tertiary structures with different amino acid compositions. Position Specific Scoring Matrix (PSSM) profiles, which have been extensively utilized in protein domain finding, secondary structure prediction, subcellular localization and other biological problems [32–34], was adopted with evolutionary significance. As presented in Figure S2 (Additional file 3), the PSSM profiles were obtained by PSI-BLAST [35] against non-redundant set of ubiquitylated protein sequences. Then, matrices of (2*n* + 1) × 20 elements, with rows that were centered at ubiquitylation sites and non-ubiquitylation sites, were extracted from PSSM profiles. Then, the (2*n* + 1) × 20 matrix was transformed into a 20 × 20 matrix by summing up the rows that were associated with the same type of amino acid. Finally, every element in 20 × 20 matrix was divided by the window length 2*n* + 1 and normalized using the expression: $\frac{1}{1+{e}^{-x}}$.

It has been reported that a side-chain of amino acid that undergoes post-translational modification prefers to be accessible on the surface of a protein [36]. Thus, the solvent-accessible surface area (SASA) was used to evaluate the ability of predicting ubiquitylation sites. In this investigation, the RVP-Net [37, 38] was applied to compute the ASA value from the full-length protein sequence. The computed ASA was the percentage of the solvent-accessible area of each amino acid on the protein. The ASA values of amino acids around the ubiquitylation and non-ubiquitylation sites were extracted and normalized to be between zero and one. In the investigation of secondary structure, version 2.0 of PSIPRED [39] was employed to compute the secondary structure from the protein sequence. The output of PSIPRED is given in terms of “H,” “E” and “C” which represent helix, sheet and coil, respectively. The full-length protein sequences with ubiquitylated lysines were submitted to PSIPRED to obtain the secondary structure of all residues. Then, an orthogonal binary coding scheme was used to transform the three types of secondary structure into numeric vectors: “100” for helix, “010” for sheet, and “001” for coil structure.

### Model building and performance evaluation

Based on binary classification, the positive and negative datasets were used to build a predictive model using support vector machine (SVM). We employed a public SVM library, LIBSVM [40], to implement the construction of predictive model and the evaluation of prediction performance. The radial basis function (RBF):

1 $$ K\left({S}_i,{S}_j\right)= \exp \left(-\gamma {\left\Vert {S}_i-{S}_j\right\Vert}^2\right) $$

was adopted as the kernel function for transforming the input samples into a higher dimensional space, and finding a hyper-plane to discriminate between the two classes with maximal margin and minimal error. The power of RBF kernel could be determined by tuning the gamma (*g*) parameter, while the cost (*c*) parameter controls the hyper-plane softness. In this work, each feature was adopted to generate a predictive model by LIBSVM library; then, the feature performing best was selected as the final feature to implement the prediction tool.

In the evaluation of predictive power, the five-fold cross-validation was carried out for each SVM model trained with different feature to test their predictive performances. The method of cross-validation could increase the reliability of performance evaluation, because it considers all original data in both the training and testing data sets; typically, each data was regarded as a test set only once [41]. To gauge the predictive performance of training model, the following measures were used: sensitivity (Sn), specificity (Sp), accuracy (Acc) and Matthews Correlation Coefficient (MCC):

2 $$ \mathrm{S}\mathrm{n} = \frac{\mathrm{TP}}{\mathrm{TP}+\mathrm{F}\mathrm{N}} $$

3 $$ \mathrm{S}\mathrm{p}=\frac{\mathrm{TN}}{\mathrm{TN}=\mathrm{F}\mathrm{P}} $$

4 $$ \mathrm{A}\mathrm{c}\mathrm{c} = \frac{\mathrm{TP}+\mathrm{T}\mathrm{N}}{\mathrm{TP}+\mathrm{F}\mathrm{P}+\mathrm{T}\mathrm{N}+\mathrm{F}\mathrm{N}} $$

5 $$ \mathrm{M}\mathrm{C}\mathrm{C}=\frac{\left(TP\times TN\right)-\left(FN\times FP\right)}{\sqrt{\left(TP+FN\right)\times \left(TN+FP\right)\times \left(TP+FP\right)\times \left(TN+FN\right)}} $$

where TP, TN, FP and FN represent the numbers of true positives, true negatives, false positives and false negatives, respectively. Sensitivity is the percentage of true positive predictions, while specificity represents that of true negative predictions. Accuracy reflects the overall proportion of true positive and true negative predictions. As for binary classifications, accuracy is sometimes not useful when the two classes are of very different sizes. Accordingly, the MCC is typically considered as a balanced measure, even if the two classes are of very different sizes [42]. The MCC value is ranging from −1 to +1: a coefficient value of +1 represents a perfect prediction, while the values 0 and −1 represent random and opposite predictions, respectively. In this work, the model containing a higher positive MCC value indicates a better prediction performance for classifying positive and negative data correctly.

### Discovery of substrate motifs of ubiquitin-conjugation sites

Along the protein ubiquitylation pathway, E3 ubiquitin ligase has a critical role in catalyzing the attachment of ubiquitin to lysine residue of a protein by recognizing a specific substrate site. Owing to an increasing number of large-scale ubiquitylation data obtained from mass spectrometry-based proteomics, we are motivated to explore potential substrate motifs for ubiquitin-conjugated sites. Although the WebLogo [43] can display position-specific amino acid composition for a group of aligned signal sequences, it is difficult to explore conserved motifs for large-scale sequence data. For instance, a sequence logo for all phosphorylation data involved with various catalytic kinases fails to obviously present the kinase-specific substrate specificity. Hence, the MDDLogo [29] was applied to the training data in an attempt to discover substrate motif signatures at ubiquitylation sites. Previous works [44–55] have demonstrated the effectiveness of dividing a group of protein sequences into smaller subgroups before the computational identification of PTM sites. MDDLogo applies chi-square test to evaluate the dependence of the occurrence of amino acids between two positions, *A*_*i*_ and *A*_*j*_, which are adjacent to the modification site (Figure S3 in Additional file 4). After the recursive chi-square test, MDDLogo divides a group of aligned sequences into subsets that capture the most significant dependencies of positions on each other. When applying MDDLogo, a parameter, i.e., the maximum-cluster-size, should be set. If the size of a subgroup is less than a specified value of maximum-cluster-size, the subgroup will not be divided any further. The MDDLogo terminates after all of the subgroup sizes are less than the value of the specified value of maximum-cluster-size.

### Construction of two-layered predictive model

**Fig. 1:**
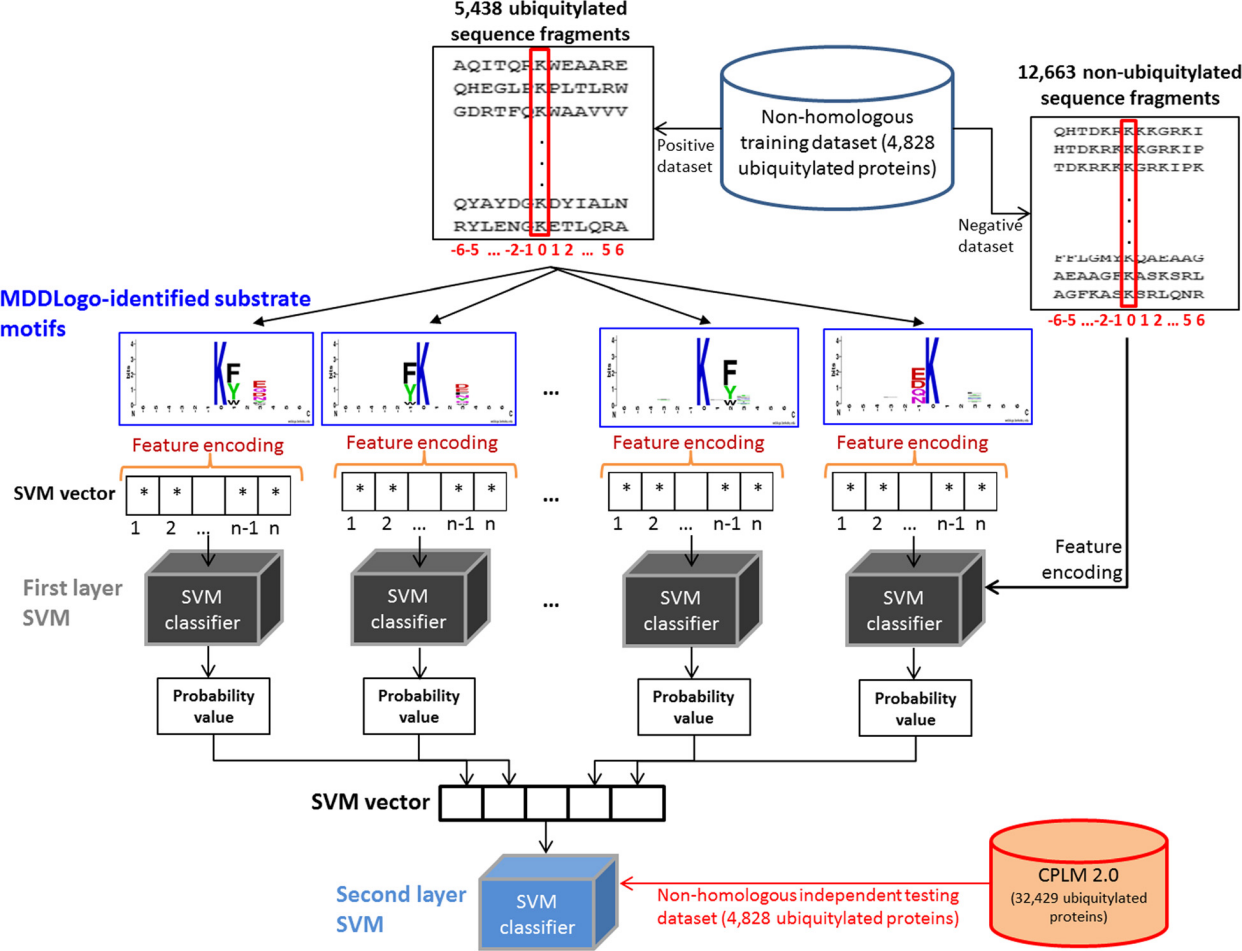
Flowchart of constructing two-layered prediction model based on MDDLogo-identified substrate motifs

In this investigation MDDLogo was utilized to sub-divide 5438 ubiquitylated sequence fragments (positive training data) into 12 subgroups that contain significant substrate motifs. As presented in Fig. 1, the LIBSVM was employed to generate first-layered SVM model for each MDDLogo-identified substrate motifs. The negative data for each MDDLogo-clustered subgroup were selected from the negative training data (12,663 non-ubiquitylated sequences) with a ratio of approximately 1:2.33 (which almost equals the ratio of the number of positive data to the number of negative data, 5438:12663). In first layer, each SVM model will output a value of probability estimate ranging from 0 to 1 for each prediction. Thus, the values of probability estimates from each SVM classifier trained according to a specific motif were adopted to form an input vector for second-layered SVM classifier. The so-called two-layered prediction model could be used to identify ubiquitin-conjugation sites along with their corresponding substrate motifs.

### Construction of independent testing dataset

In the prediction of PTM sites, after selecting the best predictive model with the highest MCC value in cross-validation process, an independent testing dataset, which was definitely blind to the training dataset, was generated to evaluate the real determinant of the predictive performance of the selected model. The dataset for independent testing was collected from version 2.0 of CPLM [17], which is a comprehensive database of protein lysine modifications. The positive and negative testing datasets were generated using the same method as was applied to the training dataset. The program cd-hit-2d was used with a sequence identity cutoff of 30 % to remove homologous sequences between independent testing set and training set, yielding the final independent testing dataset that contained 3732 positive and 10,664 negative data (Table 1). The testing dataset was also used to evaluate the predictive power of other prediction tools, which were compared with our method in terms of predictive performance.

## Results and discussion

### Investigation of amino acid composition and structural characteristics neighboring with ubiquitin-conjugation sites

**Fig. 2:**
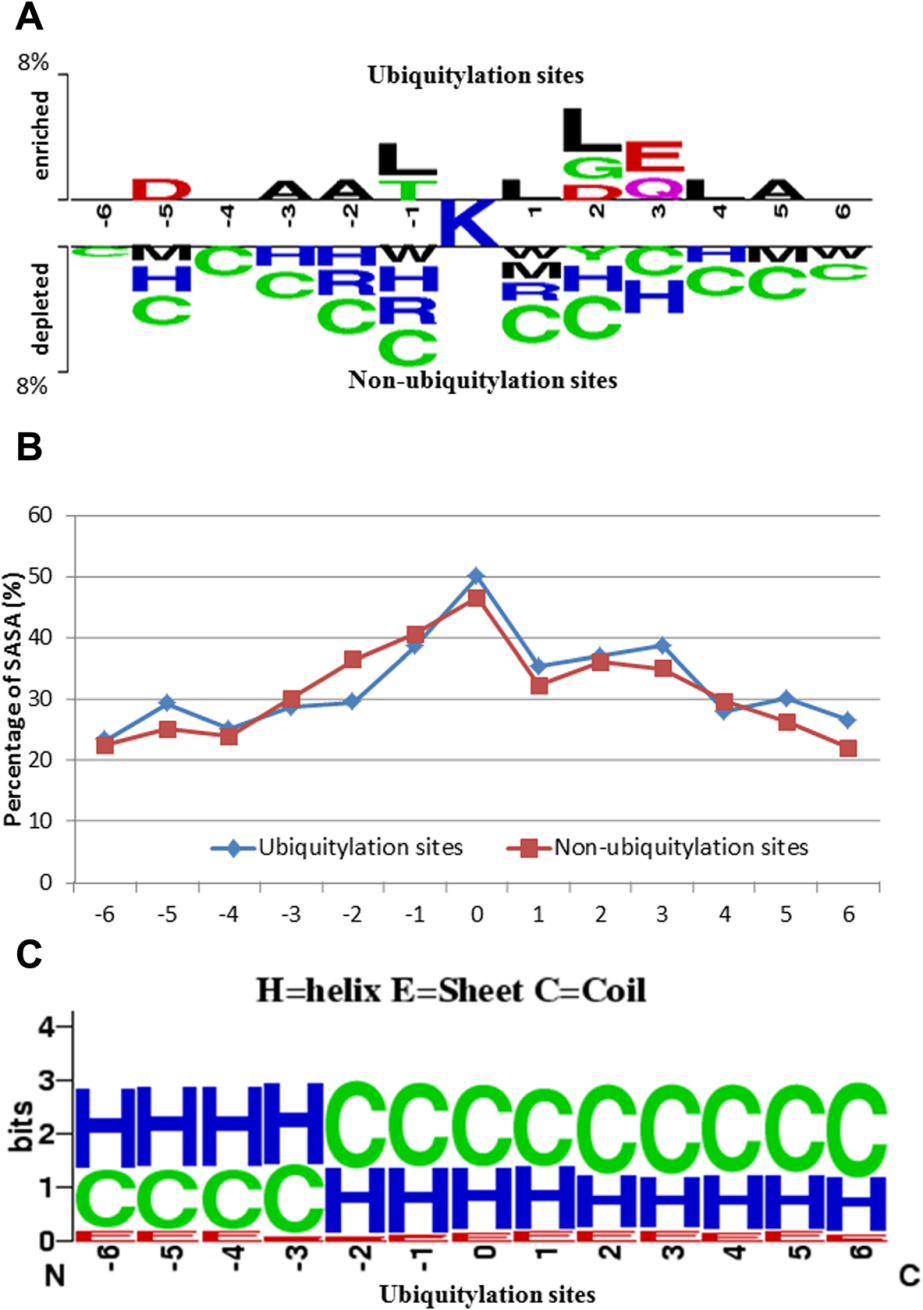
Sequenced and structural characteristics of ubiquitin-conjugation sites. **a** Comparison of position-specific amino acid composition between ubiquitylation and non-ubiquitylation sites. **b** Comparison of solvent-accessible surface area between ubiquitylation and non-ubiquitylation sites. **c** Distribution of secondary structure around ubiquitylation sites

Based on the analysis of amino acid composition, the frequency of occurrence of 20 amino acids surrounding the substrate site could be determined to find the potential consensus motifs. The flanking amino acid sequences of 5438 ubiquitylation sites (at position 0) can be graphically visualized in the entropy plots of the sequence logo by WebLogo; however, there is no conservation of amino acids around the ubiquitylation sites [14]. Hence, an effective tool, TwoSampleLogo [56], was applied to detect statistically noteworthy differences in position-specific amino acid composition between positive and negative training datasets. As presented in Fig. 2a, the lysine residue was placed in the middle of the fragment sequences, and positions of the flanking amino acids were described in range from −6 to +6. The comparison of position-specific amino acid composition between 5438 ubiquitylated and 12,663 non-ubiquitylated sites reveals that the nonpolar and aliphatic amino acids, such as alanine (A), leucine (L) and glycine (G), are enriched around ubiquitylation sites at positions −3, −2, −1, +1, +2, +4 and +5 (with *p-value* < 0.01). Additionally, negatively charged amino acids, aspartic acid (D) and glutamic acid (E), are found at positions −5, +2, and +3. By contrast, non-ubiquitylated sites have the enrichment of positively charged amino acids, histidine (H) and arginine (R) at positions −5, −3, −2, −1, +1, +2, +3 and +4. It would be noticed that a neutral amino acid, cysteine (C), is enriched around non-ubiquitylation sites; oppositely, cysteine is highly depleted in close to ubiquitylation sites. In summary, this investigation indicates a significant depletion of H, R and C residues for ubiquitylation sites.

To investigate the preference of the solvent-accessible surface area (SASA) that surrounds ubiquitin conjugation sites in protein tertiary structures, all experimentally identified ubiquitylation sites were mapped to the corresponding positions of the protein entries in the Protein Data Bank (PDB) [57]. However, only 302 ubiquitylation sites have the corresponding positions in PDB. Based on protein tertiary structures, Figure S4 (Additional file 5) indicates that the substrate sites of ubiquitin conjugation prefer to be accessible on the surface of proteins. Since most of the ubiquitylated proteins do not have corresponding protein tertiary structures in PDB, RVP-Net and PSIPRED have been adopted to compute the SASA value and secondary structure, respectively, from the protein sequence. Figure 2b presents the comparison of flanking SASA between ubiquitylation and non-ubiquitylation sites. This investigation reveals that most of the ubiquitylation sites prefer to locate in a region with higher SASA (≧35 %), especially at positions +1, +2 and +3. Overall, the mean SASA that surrounds the ubiquitylation sites slightly exceeds that around non-ubiquitylation sites. In the investigation of secondary structure, Fig. 2c indicates a higher preference of coil (loop) or helix structures around ubiquitylation sites, which is consistent with the analysis from Catic et al. [58].

### Performance evaluation of the investigated features in identifying ubiquitylation sites

To examine the effectiveness of various features, namely 20D, AAC, AAPC, PWM, PSSM, SASA and SS, in discriminating between 5438 ubiquitylation sites and 12,663 non-ubiquitylation sites, the LIBSVM was employed to learn a predictive model for each feature. As shown in Table 2, the SVM model trained with PSSM profile has the highest MCC value at 0.0.374, and relatively high sensitivity, specificity and accuracy at 69.46 %, 70.69 % and 70.32 %, respectively. Based on the large-scale dataset, the SVM model, learned from AAPC with 441-dimensional vector, also performs as best as PSSM model. In the evaluation of structural characteristics, the SVM model trained with SASA yields an acceptable performance, including 64.58 % sensitivity, 65.47 % specificity, 65.20 % accuracy, and MCC value at 0.278. On the other hand, the secondary structure (SS) is found to be the worse feature for the prediction of ubiquitylation sites, with sensitivity at 55.20 %, specificity at 60.51 %, accuracy at 58.91 %, and MCC at 0.145. In addition, the best feature (PSSM) was considered to combine with other features for obtaining a better performance in identifying ubiquitylation sites. However, the hybrid features that incorporated PSSM with other single features provide similar prediction performance with using only PSSM feature. Overall, the SVM model learned from PSSM profiles could provide best predictive performance based on the large-scale ubiquitylation data. This investigation indicates that the PSSM profile, obtained by PSI-BLAST against non-redundant set of ubiquitylated protein sequences, could reflects the evolutionary conservation of amino acids which are prone to occur in the conserved domains for ubiquitin conjugation.

**Table 2:** Performance evaluation of the investigated features in identifying ubiquitylation sites based on five-fold cross-validation

Investigated features	Sensitivity	Specificity	Accuracy	MCC
20D binary coding (20D)	65.59 %	67.09 %	66.64 %	0.303
Amino Acid Composition (AAC)	64.34 %	65.44 %	65.11 %	0.275
Amino Acid Pair Composition (AAPC)	68.70 %	70.72 %	70.11 %	0.367
Position Weight Matrix (PWM)	68.08 %	67.99 %	68.01 %	0.334
Position-Specific Scoring Matrix (PSSM)	69.46 %	70.69 %	70.32 %	0.374
Solvent-Accessible Surface Area (SASA)	64.58 %	65.47 %	65.20 %	0.278
Secondary Structure (SS)	55.20 %	60.51 %	58.91 %	0.145

In order to assess the practicability of the constructed models, an independent testing dataset was generated from CPLM database. After the removal of homologous sequences with sequence identity ≦ 30 %, the independent testing dataset is composed of 3732 positive and 10,664 negative data. Table 3 reveals that the PSSM model could yield best MCC and accuracy at 0.369 and 69.68 %, respectively, in the independent testing dataset. Interestingly, the PWM model, reaching highest sensitivity at 73.90 %, has similar prediction performance with PSSM model; otherwise, the AAPC model has the best predictive specificity at 69.05 %. Overall, the SVM model learned from PSSM profiles outperforms all others based on the large-scale independent testing dataset.

**Table 3:** Performance evaluation of the SVM models trained with various features based on independent testing dataset (3732 ubiquitylation sites and 10,664 non-ubiquitylation sites)

Training features	Sensitivity	Specificity	Accuracy	MCC
20D binary coding (20D)	62.59 %	65.85 %	65.00 %	0.253
Amino Acid Composition (AAC)	66.37 %	64.63 %	65.08 %	0.274
Amino Acid Pair Composition (AAPC)	69.05 %	69.05 %	69.05 %	0.340
Position Weight Matrix (PWM)	73.90 %	67.29 %	69.01 %	0.364
Position-Specific Scoring Matrix (PSSM)	73.20 %	68.45 %	69.68 %	0.369
Solvent-Accessible Surface Area (SASA)	63.91 %	61.36 %	62.02 %	0.223
Secondary Structure (SS)	55.60 %	51.34 %	52.45 %	0.061

### Effectiveness of incorporating substrate motifs into the identification of ubiquitylation sites

**Fig. 3:**
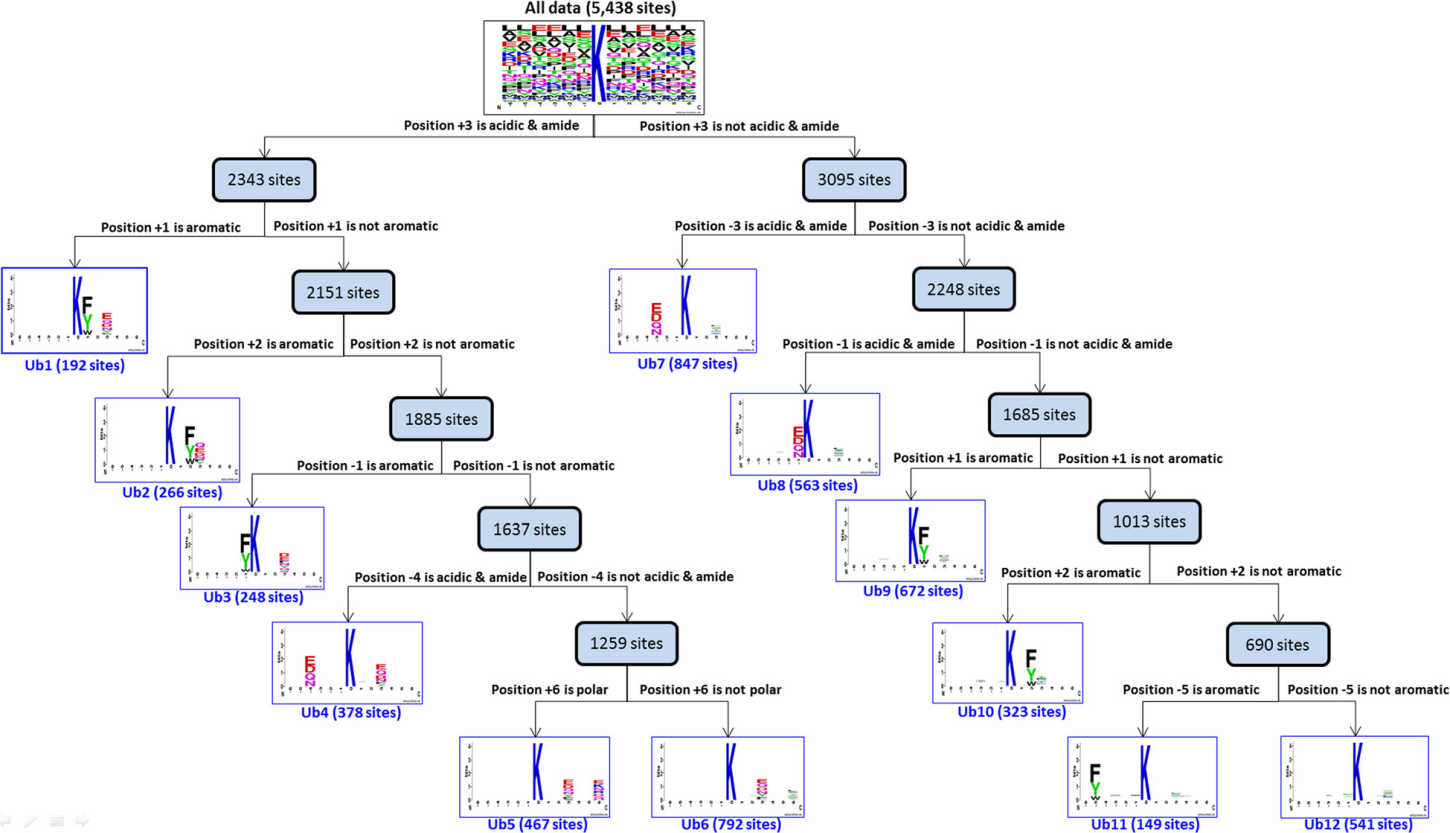
Tree view of MDDLogo-clustered subgroups with statistically significant motifs for 5438 ubiquitylation sites

In this investigation, the MDDLogo was adopted to explore the conserved motifs by dividing positive training dataset (5438 sites) into 12 subgroups. Each subgroup represents a potential ubiquitin-conjugated motif that contains statistically significant dependencies of amino acid composition between specific positions. Figure 3 provides a tree-like visualization of MDDLogo-clustered subgroups with statistically significant motifs for 5438 ubiquitylation sites. On the left subtree, six motifs (subgroups Ub1 to Ub6) out of all MDDLogo-clustered subgroups were detected based on the occurrence of acidic and amide amino acids (D, E, N and Q) at position +3, with maximal dependence value. At the same time, subgroups Ub1 (192 sites), Ub2 (266 sites) and Ub3 (248 sites) represented the occurrence of aromatic amino acids (F, Y and W) at positions +1, +2 and −1, respectively, with maximal dependence values. Subgroup Ub4 (378 sites) had another motif of acidic and amide amino acids at position −4. Additionally, subgroup Ub5 (467 sites) had the occurrence of polar amino acids at position +6, whereas subgroup Ub6 (792 sites) had no occurrence of polar amino acids at position +6. On the right subtree of Fig. 3, subgroups Ub7 (847 sites) and Ub8 (563 sites) had the motif of acidic and amide amino acids at positions −3 and −1, respectively. Subgroups Ub9 (672 sites), Ub10 (323 sites) and Ub11 (149 sites) represented the motif of aromatic amino acids at positions +1, +2 and −5, respectively, with maximal dependence values. Finally, the remaining 541 positive data resulted in the twelfth subgroup (Ub12), which contains a little conservation of amino acids at position +3.

In order to evaluate the effectiveness of MDDLogo-identified substrate motifs in identifying ubiquitylation sites, the LIBSVM was utilized to generate a predictive model for each subgroup based on PSSM feature. Table S2 (Additional file 6) presents the five-fold cross-validation performance for 12 MDDLogo-identified motifs obtained from 5438 non-homologous ubiquitylation sites. Overall, the subgroups containing motif of aromatic amino acids (F, Y and W) appeared to generate better predictive performances. For instance, the subgroup Ub2, containing the conserved motif of aromatic residues at position +2 as well as acidic and amide residues at position +3, yielded the highest MCC and accuracy at 0.881 and 94.70 %, respectively. Having similar substrate motifs with Ub2, subgroups Ub1 and Ub3 obtained predictive accuracies at 90.61 % and 89.70 %, respectively. Additionally, subgroups Ub9, Ub10 and Ub11, which contain the conserved motif of aromatic residues at specific positions, also obtained relatively high accuracies at 89.40, 91.16 and 86.04 %, respectively. In particular, subgroup Ub4, containing the conserved motif of acidic and amide residues at positions −4 and +3, yielded the second high accuracy at 92.84 %. The subgroups without clearly conserved motifs of aromatic residues generally showed relatively low accuracy (≤ 80.0 %). Subgroup Ub12, which has no conserved motif, yielded the lowest accuracy at 67.18 %.

**Fig. 4:**
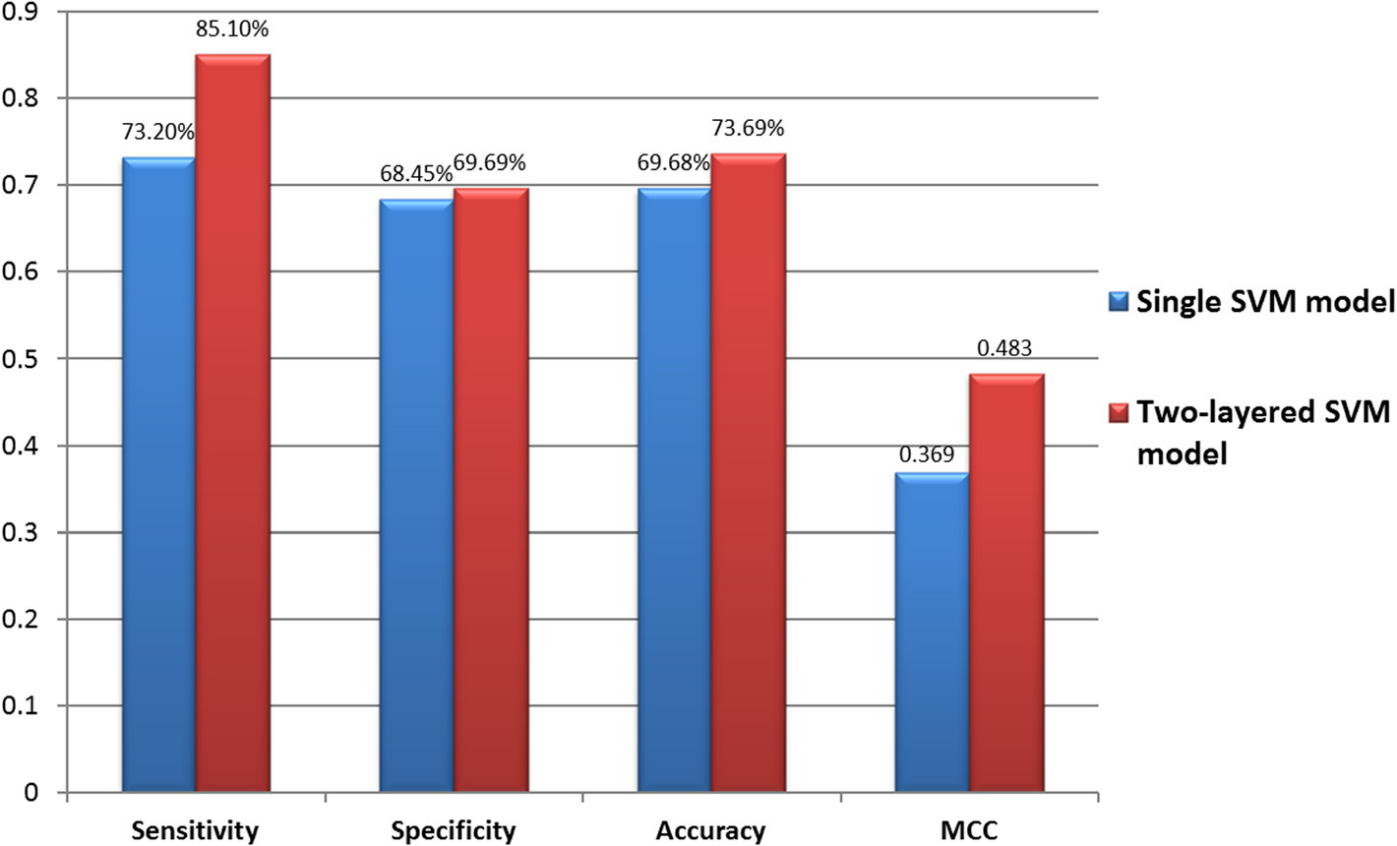
Comparison of independent testing performance between single SVM model and two-layered SVM model

In general, almost all subgroups, containing the conserved motif of aromatic amino acids at specific positions, could yield a promising accuracy. This investigation indicates that the substrate sites for ubiquitin conjugation may depend on the occurrence of aromatic residues. Table S2 (Additional file 6) shows that most of the SVM models trained with MDDLogo-identified motifs outperformed the SVM models trained with all training dataset. The mean accuracy of combining the 12 SVM models was 81.06 %, and the mean MCC was 0.586. Additionally, the independent testing dataset was used to examine the effectiveness of the two-layered SVM model, which integrates 12 MDDLogo-identified motifs, against that of single SVM model, based on the best feature (PSSM). As presented in Fig. 4, the testing results further support the effectiveness of the MDDLogo-identified motifs in the prediction of ubiquitylation sites. Moreover, the independent testing dataset was used to compare the two-layered SVM model with three available prediction tools, namely UbiPred [59], UbiProber [28] and hCKSAAP_UbiSite [22]. As shown in Table S3 (Additional file 7), the two-layered SVM model obtained highest performance when compared to others, reaching at 85.10 % sensitivity, 69.69 % specificity, 73.69 % accuracy and the 0.483 of MCC value.

### Web resource for identifying ubiquitylated lysines with substrate motifs

**Fig. 5:**
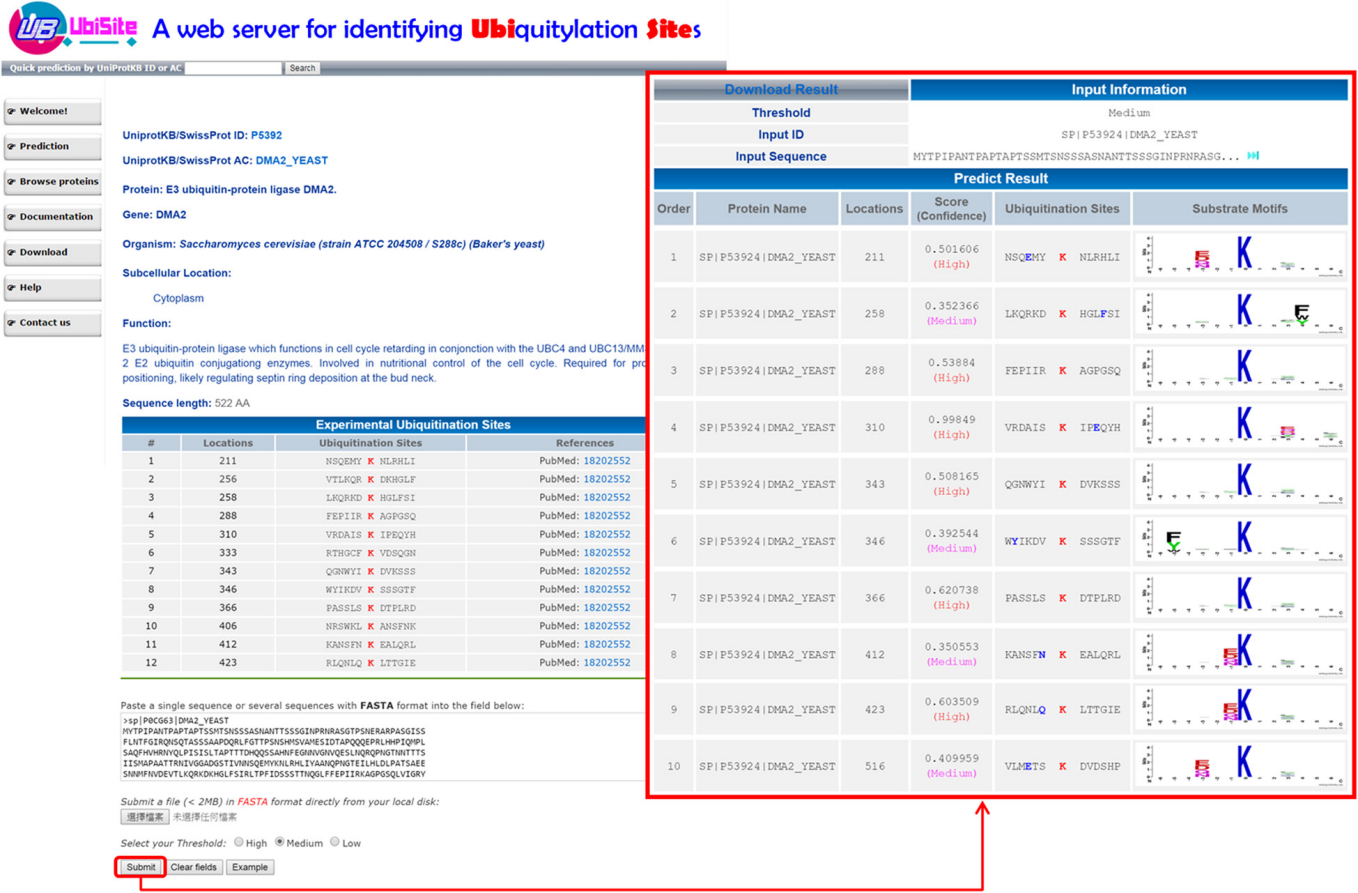
Case study of identifying ubiquitylation sites on *E3 ubiquitin-protein ligase* DMA2 of *Saccharomyces cerevisiae*

Owing to the time-consuming and lab-intensive experimental identification of site-specific ubiquitinome, a biologist may only concluded that a protein can be ubiquitylated but the precise substrate sites for ubiquitin conjugation remains unknown. Hence, an effective prediction system can help focusing efficiently on potential substrate sites. After evaluation by cross-validation and independent testing, the two-layered SVM model incorporating 12 MDDLogo-identified substrate motifs has been adopted to develop a web resource, named UbiSite, for identifying lysine ubiquitylation sites based on MDDLogo-identified substrate motifs. UbiSite allows users to submit their protein sequences in FASTA format; then, the system returns the prediction results, including ubiquitylated positions, flanking amino acids, and corresponding substrate motifs. A case study of *E3 ubiquitin-protein ligase* DMA2 from *Saccharomyces cerevisiae*, which is not included in the training dataset, was provided to demonstrate the effectiveness of UbiSite. As presented in Fig. 5, the DMA2 had 12 experimentally confirmed ubiquitylation sites located at positions 211, 256, 258, 288, 310, 333, 343, 346, 366, 406, 412 and 423 [60]. UbiSite predicted ten ubiquitylation sites for the protein sequence of DMA2, at positions 211, 258, 288, 310, 343, 346, 366, 412, 423 and 516, as well as their corresponding substrate motifs. The position 516 is a false positive prediction resulting in the predictive accuracy of 90.0 %. Another case study regards to the prediction on *tumor antigen p53* (TP53) in *Homo sapiens* (Human). TP53 has 11 ubiquitylation sites at positions 101, 120, 132, 164, 291, 292, 305, 320, 321, 357 and 370 [15, 61, 62]. As shown in Figure S5 (Additional file 8), UbiSite identified 11 ubiquitylation sites at positions 101, 120, 291, 292, 305, 319, 320, 321, 351, 357 and 382, with corresponding substrate motifs. Positions 319, 351 and 382 are false positive predictions, while positions 132, 164 and 370 are false negative predictions.

## Conclusion

In proteomics, understanding the mechanisms that underlie ubiquitylation and ubiquitin binding specificity is both essential and challenging. The high throughput of mass spectrometry-based proteomics has provided an opportunity to explore the substrate motifs for ubiquitin conjugation, based on large-scale ubiquitinome dataset. Through TwoSampleLogo, the analysis of position-specific amino acids composition between ubiquitylation and non-ubiquitylation sites revealed the significant dependencies of flanking amino acids around the substrate sites. This investigation also found that the solvent-accessible surface area of amino acids surrounding ubiquitylation sites have a tendency to higher than that around non-ubiquitylation sites. Based on the results of five-fold cross-validation, the PSSM, which reflected the evolutionary conservation of amino acids in the domain of ubiquitin conjugation, was estimated as the best feature with the highest proportion of sensitivity, specificity, accuracy and MCC value. By using MDDLogo, all ubiquitylated sequences were clustered into 12 subgroups corresponding with 12 conserved motifs. The 12 identified motifs can thus be adopted to construct a two-layered SVM model to identify ubiquitylation sites based on large-scale ubiquitylation dataset. As expected, the two-layered SVM model, which combined 12 MDDLogo-identified substrate motifs, could yield the best performance in discriminating between ubiquitylation and non-ubiquitylation sites. Consequently, the two-layered SVM model was employed to set up a web-based resource, named UbiSite, to identify ubiquitylation sites and their corresponding substrate motifs.

### Availability

The proposed method is implemented as a web-based resource, which is now freely available to all interested users at http://csb.cse.yzu.edu.tw/UbiSite/. All of the data set used in this work is also available for download in the website.

## Additional files

Additional file 1: Figure S1. The HECT-type structure of E3 ligase. (DOCX 172 kb) Additional file 2: Table S1. Data statistics of using CD-HIT with various parameters of sequence identity. (DOCX 15 kb) Additional file 3: Figure S2. Detailed steps for obtaining the PSSM vector in this study. (DOCX 768 kb) Additional file 4: Figure S3. Flowchart of MDDLogo analysis on ubiquitylated sequences. (DOCX 385 kb) Additional file 5: Figure S4. Solvent-accessible surface area around ubiquitylated lysines based on protein tertiary structures. (DOCX 70 kb) Additional file 6: Table S2. The five-fold cross-validation performance for 12 MDDLogo-identified motifs obtained from 5438 non-homologous ubiquitylation sites. (DOCX 93 kb) Additional file 7: Table S3. Comparison of predictive performance between the presented models and other prediction tools based on independent testing dataset (3732 ubiquitylation sites and 10,664 non-ubiquitylation sites). (DOCX 16 kb) Additional file 8: Figure S5. Case study of identifying ubiquitylation sites on *tumor antigen p53* (TP53) in *Homo sapiens* (Human). (DOCX 713 kb)
