# Supplementary material for: UbiSite: incorporating two-layered machine learning method with substrate motifs to predict ubiquitin-conjugation site on lysines
Source: BMC Syst Biol. 2016 Jan 11;10(Suppl 1):6. doi: 10.1186/s12918-015-0246-z (PMC4895383; doi:10.1186/s12918-015-0246-z)
Supplement: Additional file 1: Figure S1. — The HECT-type structure of E3 ligase. (DOCX 172 kb) [file 12918_2015_246_MOESM1_ESM.docx]

**
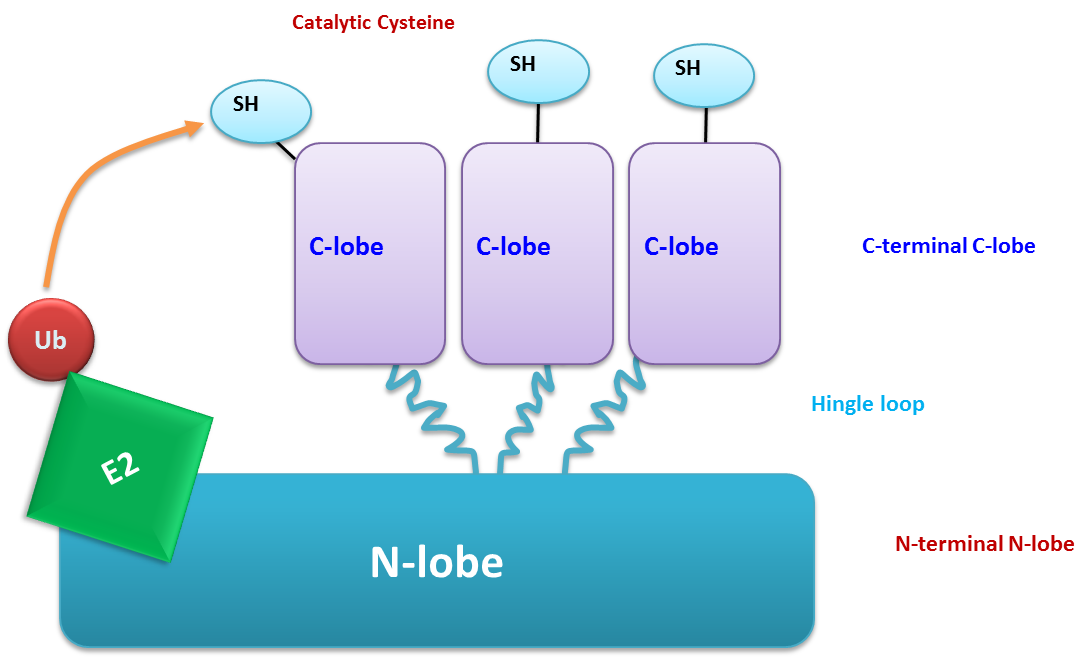
**

**Figure S1. The HECT-type structure of E3 ligase.** The HECT domain of E3 ligases plays prominent roles in trafficking, the immune response, and in several other signaling pathways that regulate cellular growth and proliferation ([Rotin and Kumar, 2009](#_ENREF_39)). The conserved HECT domain comprises approximately 350 amino acids. The HECT domain consists of two major components: a N-terminal N-lobe that interacts with the E2, and a C-terminal C-lobe which contains the active-site cysteine forming the thioester linkage with ubiquitin ([Huang, et al., 1999](#_ENREF_17); [Lin, et al., 2012](#_ENREF_25); [Metzger, et al., 2012](#_ENREF_28)). The conserved HECT domain is located at the C-terminus of these enzymes, whereas their N-terminal domains are diverse and mediate substrate targeting. Studies on HECT domains and their crystal structure suggested that these two lobes (N-lobe and C-lobe) are connected via a flexible hinge that allows them to come together during ubiquitin transfer.
