# Supplementary material for: UbiSite: incorporating two-layered machine learning method with substrate motifs to predict ubiquitin-conjugation site on lysines
Source: BMC Syst Biol. 2016 Jan 11;10(Suppl 1):6. doi: 10.1186/s12918-015-0246-z (PMC4895383; doi:10.1186/s12918-015-0246-z)
Supplement: Additional file 2: Table S1. — Data statistics of using CD-HIT with various parameters of sequence identity. (DOCX 15 kb) [file 12918_2015_246_MOESM2_ESM.docx]

**Table S1**. **Data statistics of using CD-HIT with various parameters of sequence identity.**

| **Sequence identity** | **Training set** (37,647 proteins) | | **Independent testing set** (32,429 proteins) | |
| --- | --- | --- | --- | --- |
|  | **Positive data** | **Negative data** | **Positive data** | **Negative data** |
| Original dataset | 128,026 | 1,317,734 | 139,950 | 1,109,432 |
| 90% | 119,552 | 1,197,052 | 118,232 | 978,864 |
| 80% | 102,197 | 899,692 | 96,648 | 814,739 |
| 70% | 83,887 | 674,564 | 80,229 | 581,144 |
| 60% | 56,952 | 335,961 | 47,387 | 289,249 |
| 50% | 32,999 | 212,427 | 26,612 | 199,004 |
| 40% | 19,006 | 115,452 | 14,211 | 98,772 |
| **30%** | **5,438** | **12,663** | **3,732** | **10,664** |
