# Supplementary figures and images for: UbiSite: incorporating two-layered machine learning method with substrate motifs to predict ubiquitin-conjugation site on lysines
Source: BMC Syst Biol. 2016 Jan 11;10(Suppl 1):6. doi: 10.1186/s12918-015-0246-z (PMC4895383; doi:10.1186/s12918-015-0246-z)

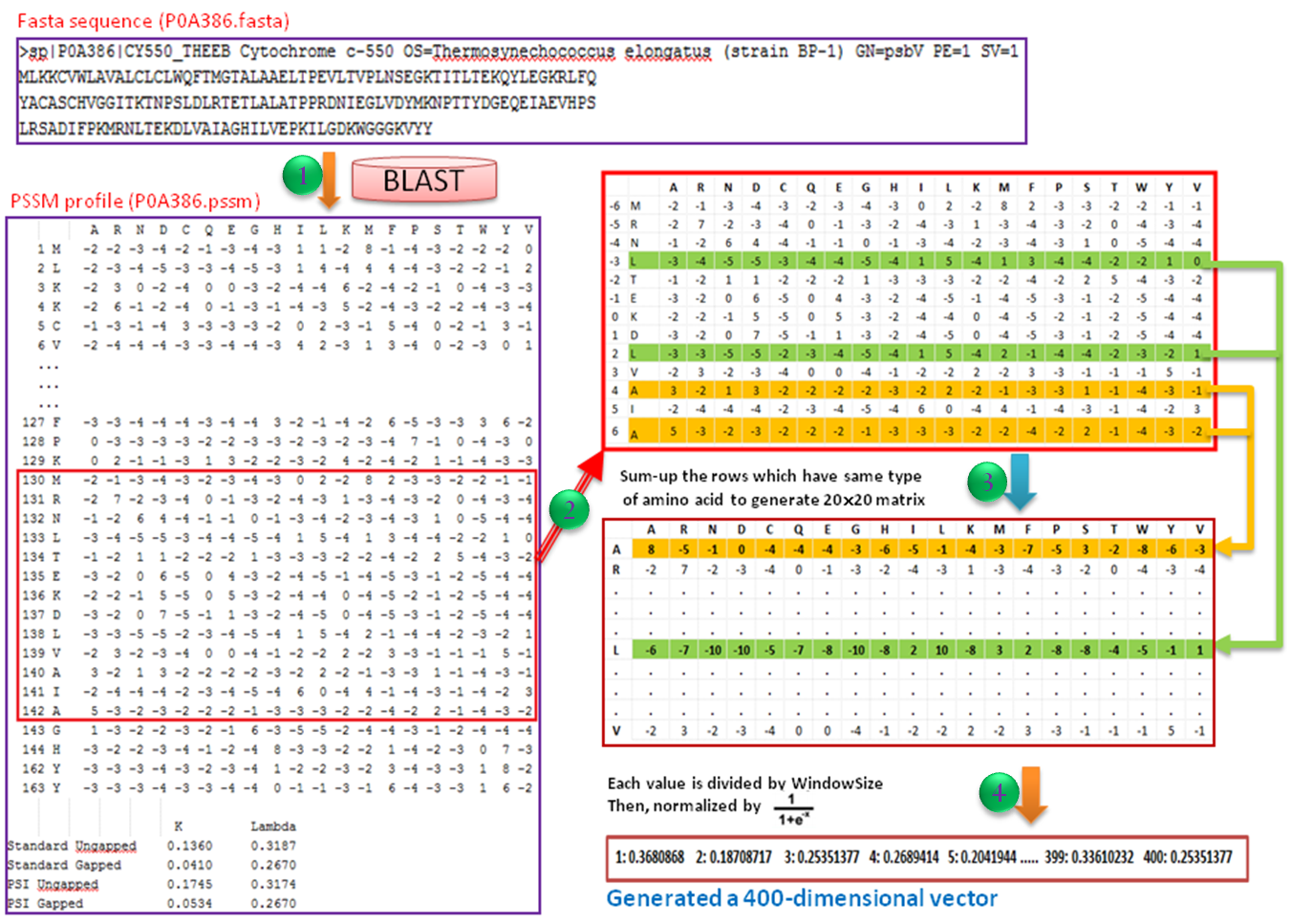


Figure S2. Detailed steps for obtaining the PSSM vector in this study.

Supplement: Additional file 3: Figure S2. — Detailed steps for obtaining the PSSM vector in this study. (DOCX 768 kb) [file 12918_2015_246_MOESM3_ESM.docx]

**
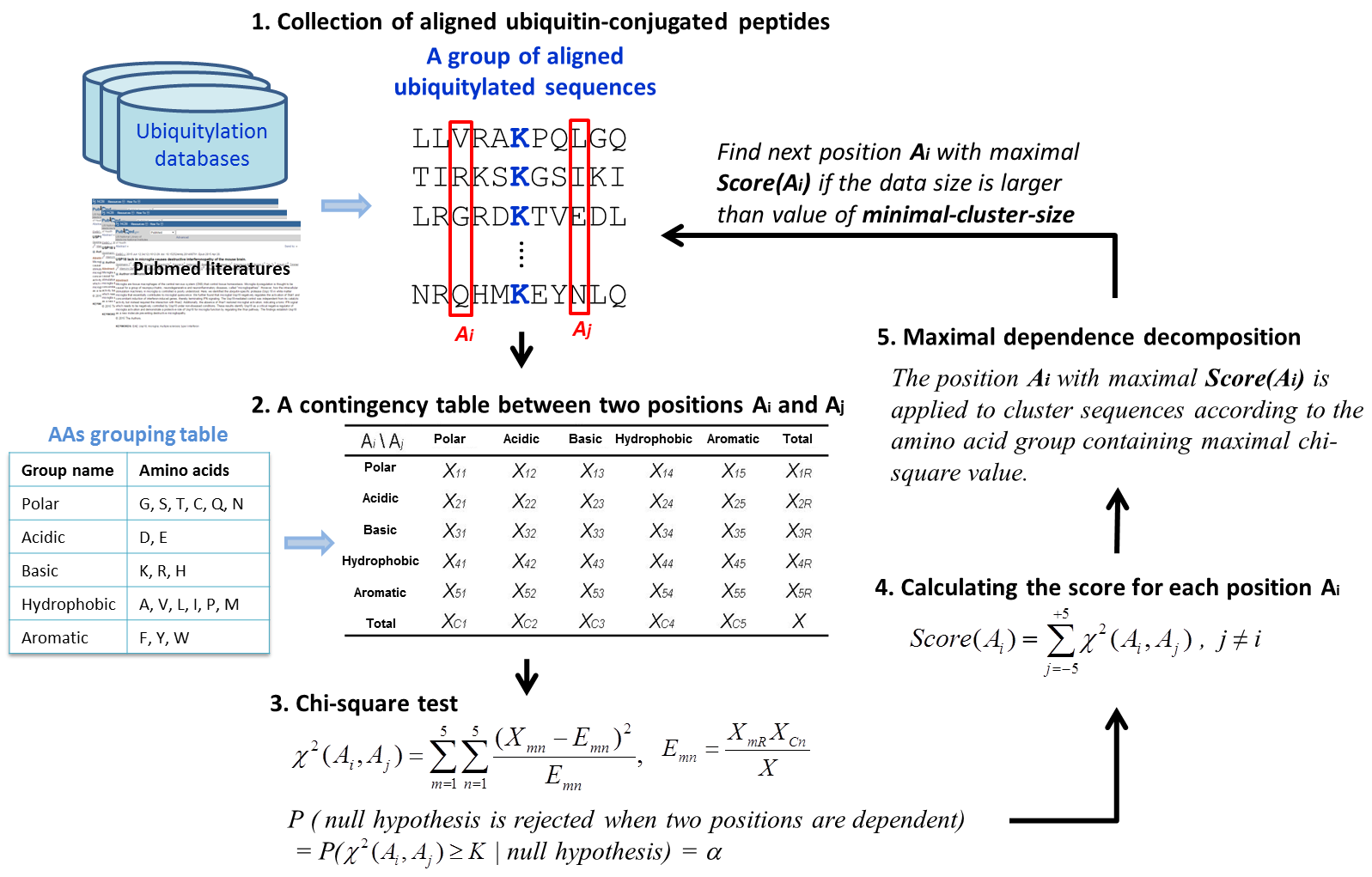
**

**Figure S3. Flowchart of MDDLogo analysis on ubiquitylated sequences.**

Supplement: Additional file 4: Figure S3. — Flowchart of MDDLogo analysis on ubiquitylated sequences. (DOCX 385 kb) [file 12918_2015_246_MOESM4_ESM.docx]
