# Supplementary material for: UbiSite: incorporating two-layered machine learning method with substrate motifs to predict ubiquitin-conjugation site on lysines
Source: BMC Syst Biol. 2016 Jan 11;10(Suppl 1):6. doi: 10.1186/s12918-015-0246-z (PMC4895383; doi:10.1186/s12918-015-0246-z)
Supplement: Additional file 5: Figure S4. — Solvent-accessible surface area around ubiquitylated lysines based on protein tertiary structures. (DOCX 70 kb) [file 12918_2015_246_MOESM5_ESM.docx]

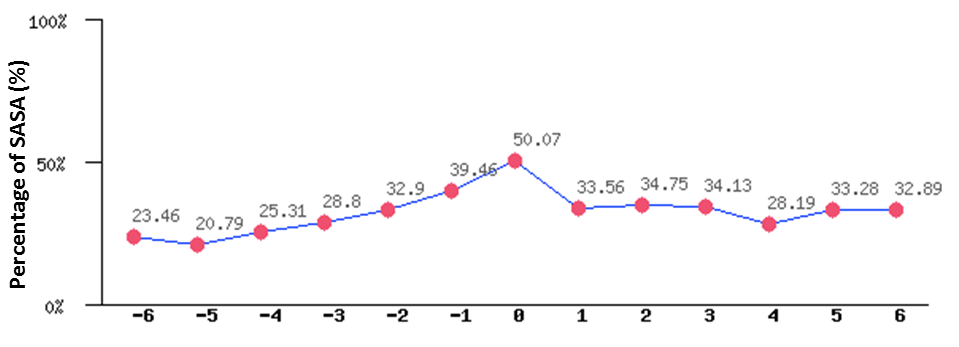


**Figure S4. Solvent-accessible surface area around ubiquitylated lysines based on protein tertiary structures.**
