# Supplementary material for: UbiSite: incorporating two-layered machine learning method with substrate motifs to predict ubiquitin-conjugation site on lysines
Source: BMC Syst Biol. 2016 Jan 11;10(Suppl 1):6. doi: 10.1186/s12918-015-0246-z (PMC4895383; doi:10.1186/s12918-015-0246-z)
Supplement: Additional file 6: Table S2. — The five-fold cross-validation performance for 12 MDDLogo-identified motifs obtained from 5438 non-homologous ubiquitylation sites. (DOCX 93 kb) [file 12918_2015_246_MOESM6_ESM.docx]

**Table S2. The five-fold cross-validation performance for 12 MDDLogo-identified motifs obtained from 5,438 non-homologous ubiquitylation sites**.

| **Subgroup** | **Number of**  **ubiquitylation sites** | **MDDLogo-identified substrate motif** | **Number of**  **non-ubiquitylation sites** | **Sensitivity** | **Specificity** | **Accuracy** | **MCC** |
| --- | --- | --- | --- | --- | --- | --- | --- |
| Ub1 | 192 | 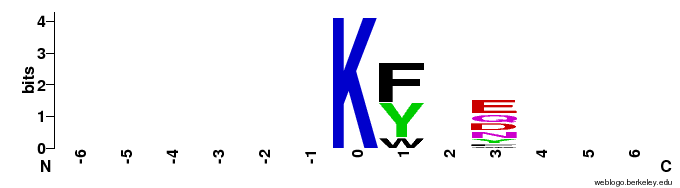 | 447 | 93.23% | 89.48% | 90.61% | 0.793 |
| Ub2 | 266 | 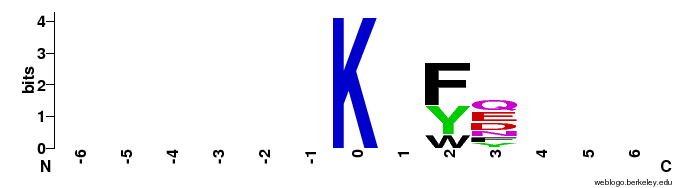 | 620 | 97.37% | 93.55% | 94.70% | 0.881 |
| Ub3 | 248 | 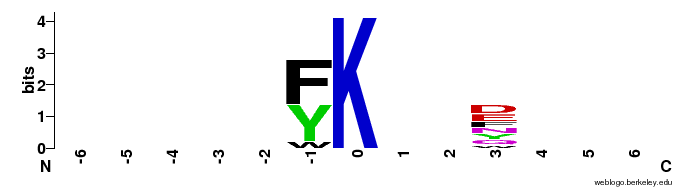 | 577 | 88.71% | 90.12% | 89.70% | 0.765 |
| Ub4 | 378 | 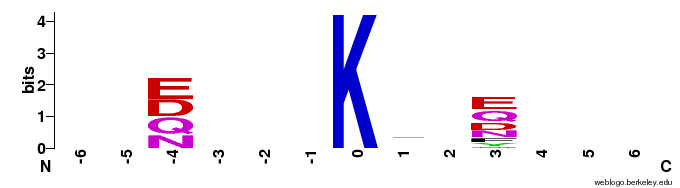 | 880 | 92.06% | 93.18% | 92.84% | 0.835 |
| Ub5 | 467 | 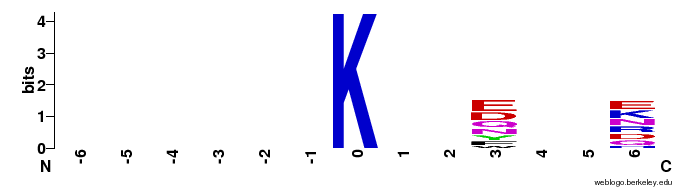 | 1,087 | 72.81% | 77.00% | 75.74% | 0.471 |
| Ub6 | 792 | 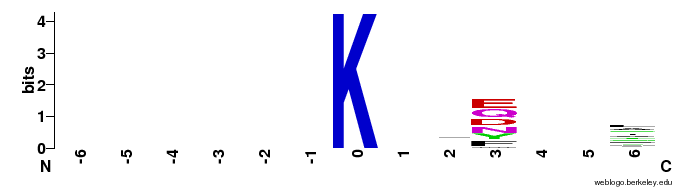 | 1,844 | 69.70% | 70.50% | 70.26% | 0.374 |
| Ub7 | 847 | 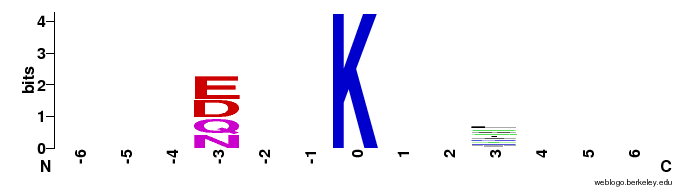 | 1,972 | 76.38% | 79.72% | 78.72% | 0.532 |
| Ub8 | 563 | 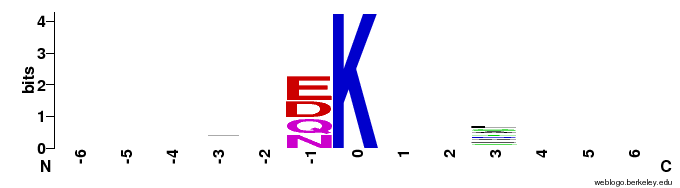 | 1,311 | 83.48% | 77.11% | 79.03% | 0.565 |
| Ub9 | 672 | 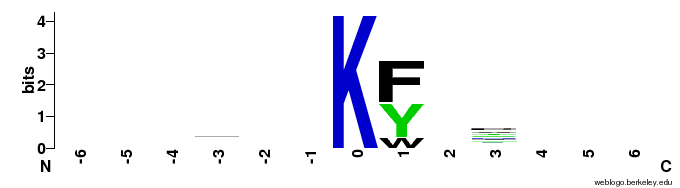 | 1,565 | 89.28% | 89.45% | 89.40% | 0.761 |
| Ub10 | 323 | 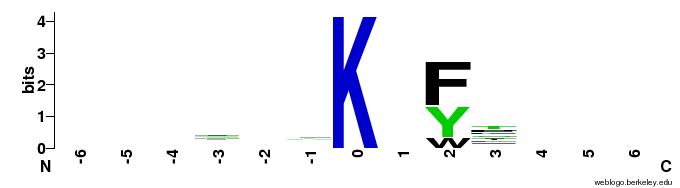 | 752 | 86.69% | 93.08% | 91.16% | 0.791 |
| Ub11 | 149 | 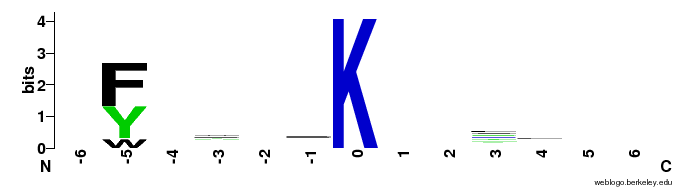 | 348 | 85.61% | 86.21% | 86.04% | 0.683 |
| Ub12 | 541 | 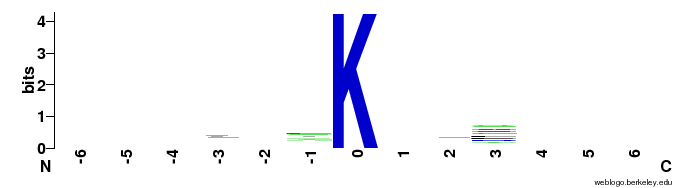 | 1260 | 66.54% | 67.46% | 67.18% | 0.315 |
| **Overall prediction performance** | | | | **80.58%** | **81.26%** | **81.06%** | **0.586** |
