# Supplementary material for: UbiSite: incorporating two-layered machine learning method with substrate motifs to predict ubiquitin-conjugation site on lysines
Source: BMC Syst Biol. 2016 Jan 11;10(Suppl 1):6. doi: 10.1186/s12918-015-0246-z (PMC4895383; doi:10.1186/s12918-015-0246-z)
Supplement: Additional file 7: Table S3. — Comparison of predictive performance between the presented models and other prediction tools based on independent testing dataset (3732 ubiquitylation sites and 10,664 non-ubiquitylation sites). (DOCX 16 kb) [file 12918_2015_246_MOESM7_ESM.docx]

**Table S3. Comparison of predictive performance between the presented models and other prediction tools based on independent testing dataset (3,732 ubiquitylation sites and 10,664 non-ubiquitylation sites).**

| **Methods** | **Training features** | **Sensitivity** | **Specificity** | **Accuracy** | **MCC** |
| --- | --- | --- | --- | --- | --- |
| **UbiPred** | Physicochemical properties | 47.32% | 47.56% | 47.50% | -0.045 |
| **UbiProber** | KNN, PCP, AAC | 71.46% | 52.24% | 57.22% | 0.208 |
| **hCKSAAP_UbSite** | CKSAAP | 23.47% | 81.22% | 66.25% | 0.051 |
| **Single SVM model** | PSSM | 73.20% | 68.45% | 69.68% | 0.369 |
| **Two-layered SVM model** | **PSSM + 12 MDDLogo-identified motifs** | **85.10%** | **69.69%** | **73.69%** | **0.483** |
