# Supplementary material for: UbiSite: incorporating two-layered machine learning method with substrate motifs to predict ubiquitin-conjugation site on lysines
Source: BMC Syst Biol. 2016 Jan 11;10(Suppl 1):6. doi: 10.1186/s12918-015-0246-z (PMC4895383; doi:10.1186/s12918-015-0246-z)
Supplement: Additional file 8: Figure S5. — Case study of identifying ubiquitylation sites on tumor antigen p53 (TP53) in Homo sapiens (Human). (DOCX 713 kb) [file 12918_2015_246_MOESM8_ESM.docx]

**
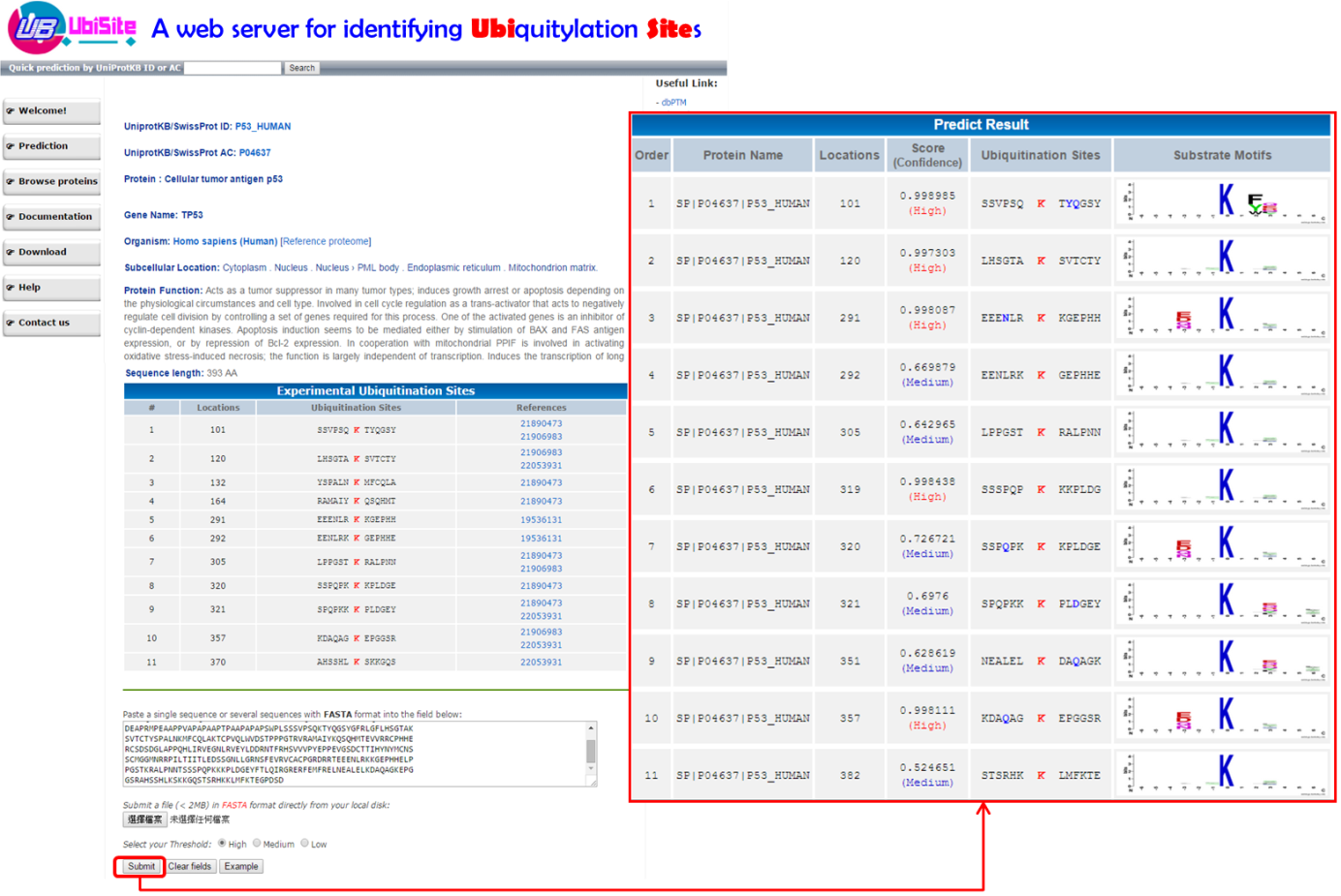
**

**Figure S5. Case study of identifying ubiquitylation sites on *tumor antigen p53* (TP53) in *Homo sapiens* (Human).**
